# Millennium development health metrics: where do Africa’s children and women of childbearing age live?

**DOI:** 10.1186/1478-7954-11-11

**Published:** 2013-07-23

**Authors:** Andrew J Tatem, Andres J Garcia, Robert W Snow, Abdisalan M Noor, Andrea E Gaughan, Marius Gilbert, Catherine Linard

**Affiliations:** 1 Department of Geography and Environment, University of Southampton, Highfield, Southampton, UK; 2 Fogarty International Center, National Institutes of Health, Bethesda, MD 20892, USA; 3 Department of Geography, University of Florida, Gainesville, Florida, USA; 4 Emerging Pathogens Institute, University of Florida, Gainesville, Florida, USA; 5 Malaria Public Health and Epidemiology Group, Centre for Geographic Medicine, KEMRI - Univ. Oxford - Wellcome Trust Collaborative Programme, Nairobi, Kenya; 6 Centre for Tropical Medicine, Nuffield Department of Clinical Medicine, University of Oxford, CCVTM, Oxford, UK; 7 Biological Control and Spatial Ecology, Université Libre de Bruxelles, Brussels, Belgium; 8 Fonds National de la Recherche Scientifique (F.R.S./FNRS), Brussels, Belgium

**Keywords:** Population, Demography, Mapping, Millenium development goals

## Abstract

The Millennium Development Goals (MDGs) have prompted an expansion in approaches to deriving health metrics to measure progress toward their achievement. Accurate measurements should take into account the high degrees of spatial heterogeneity in health risks across countries, and this has prompted the development of sophisticated cartographic techniques for mapping and modeling risks. Conversion of these risks to relevant population-based metrics requires equally detailed information on the spatial distribution and attributes of the denominator populations. However, spatial information on age and sex composition over large areas is lacking, prompting many influential studies that have rigorously accounted for health risk heterogeneities to overlook the substantial demographic variations that exist subnationally and merely apply national-level adjustments.

Here we outline the development of high resolution age- and sex-structured spatial population datasets for Africa in 2000-2015 built from over a million measurements from more than 20,000 subnational units, increasing input data detail from previous studies by over 400-fold. We analyze the large spatial variations seen within countries and across the continent for key MDG indicator groups, focusing on children under 5 and women of childbearing age, and find that substantial differences in health and development indicators can result through using only national level statistics, compared to accounting for subnational variation.

Progress toward meeting the MDGs will be measured through national-level indicators that mask substantial inequalities and heterogeneities across nations. Cartographic approaches are providing opportunities for quantitative assessments of these inequalities and the targeting of interventions, but demographic spatial datasets to support such efforts remain reliant on coarse and outdated input data for accurately locating risk groups. We have shown here that sufficient data exist to map the distribution of key vulnerable groups, and that doing so has substantial impacts on derived metrics through accounting for spatial demographic heterogeneities that exist within nations across Africa.

## Introduction

The Millennium Development Goals (MDGs) were initiated to encourage development by improving social and economic conditions in the world’s poorest countries [1]. In order to achieve this on a 15-year timeline, targets and indicators for poverty reduction and health improvement were set. There are eight goals with 21 targets, and a series of measurable indicators for each target, many of which are focused on health in specific target demographic groups, mainly children and pregnant women [1]. The initiation of these indicators, as well as a general growth in the number of health metric studies, has prompted substantial growth in approaches to measure them, with increasingly sophisticated methods that attempt to capture spatial heterogeneities in health conditions being developed (e.g. [2-10]).

An improved understanding of the geographic variation in health status and risks and access to services and care within countries is increasingly being recognized as central to meeting health and development goals and delivering equity in interventions and impacts [11-13]. For instance, approaches based on local epidemiological and coverage data have been identified as vital to achieving high impacts in reducing childhood mortality for MDGs 4 and 5 [14], while the subnational heterogeneity in HIV [15,16] and malaria [5,17] prevalences mean that effective targeting of interventions remains vital in achieving MDG 6 [1]. Indicators assessed at national scales can often conceal important inequities, with the rural poor often least well represented [12,18]. Moreover, as international funding for health and development comes under pressure, the ability to target limited resources to underserved groups becomes crucial. Substantial demographic variations exist across countries and between urban and rural areas [19]. With MDG health indicators tied to specific vulnerable groups, there is a need to know where these vulnerable groups are and the number of individuals at risk that exist in order to accurately characterize denominators.

Health metrics continue to be collected, analyzed, and reported at national scales (e.g. [20-22]); however, datasets collected at subnational levels are increasingly available, and approaches that attempt to capture the spatial heterogeneity that often exists subnationally are being developed. The importance of geography is being recognized in development [23], mortality [24], and disease risks [19,25], with methods for mapping these factors at fine subnational scales becoming increasingly sophisticated and common in large-scale health metric studies [25]. While such projects are utilizing contemporary and fine resolution datasets to build the most spatially accurate evidence bases for MDG progress tracking, each are generally combined with spatial population datasets that contain no subnational information on target demographic groups to obtain denominators [19]. This lack of spatial data to quantify age groups by sex has meant that the increasing number of studies that are mapping indicators and risks subnationally continue to rely on simple national adjustments of spatial population data to provide denominators. For example, to estimate the number of children under 5 years old living at risk of *P. falciparum* malaria in Tanzania, previous work [5] has involved the development of a detailed map of prevalence from hundreds of community prevalence surveys, then overlaying this onto a detailed gridded population distribution dataset [26] to estimate total populations at risk, but then simply using the United Nations national-level estimate [27] of the proportion of the population that is under 5 (17.9%) to convert this to an estimate of under-5s at risk, despite clear evidence of large subnational differences in the proportions of residential populations that are under 5 [19]. Further examples where similar national-level adjustments have been made include the estimation of numbers of pediatric fevers associated with malaria [28], numbers of preschool children at risk of anemia [10], schistosomiasis prevalence in children and under-20-year-olds [9,29], numbers of children residing in areas suitable for seasonal malaria chemoprevention [30], and global malaria mortality [6]. Moreover, in each of these cases, and for many other cartography-based health metric projects, the spatial demographic data used has been adjusted to a year of interest using national-level growth rates, ignoring the fact that the population distribution of a country changes heterogeneously over time [19].

Here we assess the importance of accounting for subnational demographic variations in deriving health metrics. We present the development of ~100 m spatial resolution age- and sex-structured spatial population datasets for Africa built from satellite imagery and over a million measurements derived from more than 20,000 subnational administrative units and originating from a variety of publicly available sources that include census data and national household surveys. The effects of accounting for subnational demographic heterogeneity on estimates of the numbers of women of childbearing age and children under 5 years old impacted by long travel times to services and risks of malaria transmission, respectively, are then quantified to provide illustrative analyses.

## Methods

### Constructing a detailed and contemporary population distribution dataset

The AfriPop project (http://www.afripop.org) has recently completed construction of 2010 and 2015 estimates of population distribution for continental Africa and Madagascar at approximately 100 m spatial resolution. Full details are provided in Linard *et al.*[31] and on the project website (http://www.afripop.org). Briefly, a GIS-linked database of census and official population estimate data was constructed, targeting the most recent and spatially detailed datasets available, given their importance in producing accurate mapping [31-33]. Detailed maps of settlement extents were derived from Landsat satellite imagery through either semi-automated classification approaches [33,34] or expert opinion-based analyses [31]. These settlement maps were then used to refine land cover data, while local census data mapped at fine resolution enumeration area level from sample countries across the continent were exploited to identify typical regional per-land cover class population densities, which were then applied to redistribute census counts to map human population distributions at 100 m spatial resolution continent-wide [31,33,35]. Where available, additional country-specific datasets providing valuable data on population distributions not captured by censuses, such as internally displaced people or detailed national surveys, were incorporated into the mapping process [36].

### Compiling national estimates of age and sex structures

In order to examine the effects of utilizing subnational data on age and sex structures of populations, 2010 national-level data were first obtained to provide a baseline for comparison. These were obtained from the United Nations Population Division’s World Population Prospects 2010 publication [27] and are derived from national-level demographic models built upon census data. These national-level proportions were then used to adjust the gridded population dataset described above to produce separate five-year age group gridded datasets by sex, following approaches used in many previous studies that assume demographic homogeneity across countries (e.g. [6,9,10,28,30,37]).

### Compiling subnational estimates of age and sex structures

Data on subnational population compositions from the last 20 years were obtained from a variety of sources for all mainland African countries, plus Madagascar (Additional file 1: Protocol S1). Contemporary census-based counts broken down at a fine resolution administrative unit level generally provide the most reliable source for population composition mapping, due to the large sample sizes providing reliable information summarized for small areas. Where age and sex data reported at subnational levels were available for censuses undertaken within the last two decades, these were obtained for this study (Additional file 1: Protocol S1). An addition to the aggregated full census data are large samples of household-level records derived from censuses (census microdata) that provide age and sex structure, reported generally by administrative level 1 (e.g., province) or 2 (e.g., district). Census microdata on subnational age and sex proportions by subnational regions for African countries within the last twenty years were obtained where available (Additional file 1: Protocol S1). While census data are often readily available for high-income countries, for African countries census data with subnational reporting of age and sex structure can often be either unavailable or substantially more than a decade old. Alternative national household survey data sources were therefore exploited to provide the most contemporary and spatially detailed estimates as possible of age and sex proportions, given the constraints of their sampling frameworks. Here, national household survey data on population age and sex compositions were obtained from the most recent Demographic and Health Survey (DHS), Malaria Indicator Survey (MIS), or AIDS Indicator Survey (AIS) [38], or from Multiple Indicator Cluster Surveys (MICS) [39], for all countries where such surveys have been undertaken.

Summaries of subnational population structure by sex and five-year age groupings from either full national census summaries, census microdata, or household surveys were obtained for 47 of the 50 countries in mainland Africa, plus Madagascar. Where multiple datasets from similar time periods were available, the census or census microdata were given priority for use, due to the larger sample sizes. For four countries (Libya, Eritrea, Western Sahara, and Equatorial Guinea), no subnational estimates of age and sex structures were found, and for these countries the UN national estimates and projections for the 2000-2015 period [27] were obtained and used in the mapping. The relatively small sample sizes for household survey data and census microdata compared to those from full census data mean that age and sex proportions derived from them are more uncertain. To ensure that age proportions derived from these datasets were representative of those derived from census data, instances where (i) national household surveys were undertaken in the same year or within one year of a national census and (ii) census microdata samples and the full census that each was derived from were collated and statistical comparisons undertaken, which showed consistent and strong correlations (Additional file 1: Protocol S1).

**Figure 1:**
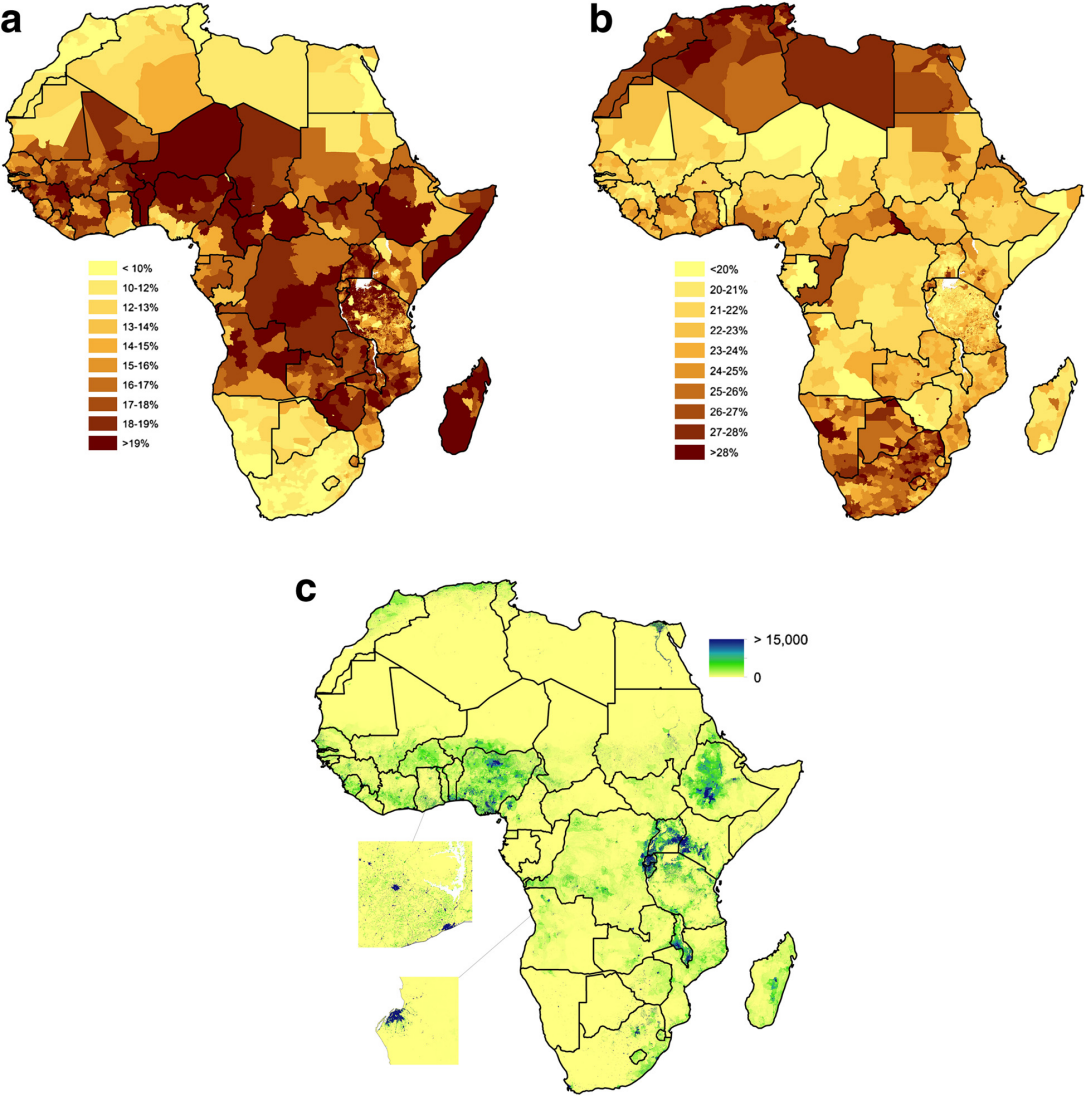
**Spatial demographic datasets for mainland Africa and Madagascar. (a)** The estimated proportion of children under 5 years old subnationally; **(b)** the estimated proportion of women of childbearing age subnationally; **(c)** the Africa-wide 1km spatial resolution gridded dataset of numbers of children under 5 years old in 2010, with close-ups showing 100m spatial resolution detail for southern Ghana and Luanda, Angola.

Once datasets on numbers and proportions of individuals by age and sex had been collated for as many subnational units as available within the last two decades, using sample weights where applicable to household surveys, these were matched to corresponding GIS datasets showing the boundaries of each unit. Africa-wide GIS-linked data on proportions of individuals by age and sex and by administrative unit were created for as close to 2010 as was available (Figure 1, further datasets are provided in Additional file 1: Protocol S1).

### Subnational and urban growth rates, projections, and adjustments

The production of spatial population datasets for Africa has previously relied on simple interpolation between census-derived timepoints where available or, more commonly, the application of UN Population Division national-level growth rate estimates [27]. For 45 of the 50 countries in mainland Africa plus Madagascar, subnational growth rates derived from either censuses or official national estimates were obtained (see Additional file 1: Protocol S1 for details). Additionally, separate growth rates for urban and rural areas nationally were obtained for those countries and time periods for which subnational growth rate data were not available [40]. Finally, estimated population sizes for named African cities [40], and the urban extents dataset used in the construction of the Global Rural Urban Mapping Project (GRUMP) [26] were obtained. The urban extents matching those African cities for which individual population totals are estimated in the UN World Urbanization Prospects [40] were identified, and the totals for 2000-2015 matched up.

### Gridded population dataset production

The GIS unit-linked age and sex subnational proportions dataset described above was used to adjust the existing AfriPop 2010 spatial population datasets [31], to produce estimates of the distributions of populations by sex and five-year age group across Africa in 2010. The datasets were then adjusted to ensure that national population totals by age group, specific city totals and urban/rural totals matched those reported by the UN [27,40]. For the analyses outlined in the remainder of this paper, the summation of the datasets representing males and females in the 0-5 year age group was undertaken to produce a 2010 distribution dataset of children under 5 years old, and the summation of datasets representing females in the 15-49 year age groups was undertaken to produce a 2010 dataset of women of childbearing age. The application of subnational growth rates to produce 2000, 2005, and 2015 datasets is described in Additional file 1: Protocol S1.

### Quantifying effects of spatial population dataset on health metrics

To examine the effects on health and development indicators through use of the new subnational characterizations of children under 5 and women of childbearing age compared to undertaking national-level age adjustments using the UN data [27], two sets of illustrative analyses were undertaken. Firstly, Africa-wide estimates of the number of children under 5 years old residing in different *Plasmodium falciparum* malaria prevalence classes were calculated, and secondly, estimates of the number of women of childbearing age residing at different travel times from the nearest major settlement (population >50,000) across Africa and nearest health facility for countries with open access geolocated datasets of facilities were estimated. In each case the focus was on the size of the change in output metrics through accounting for demographic spatial heterogeneity, rather than the estimates produced and their fidelity.

**Figure 2:**
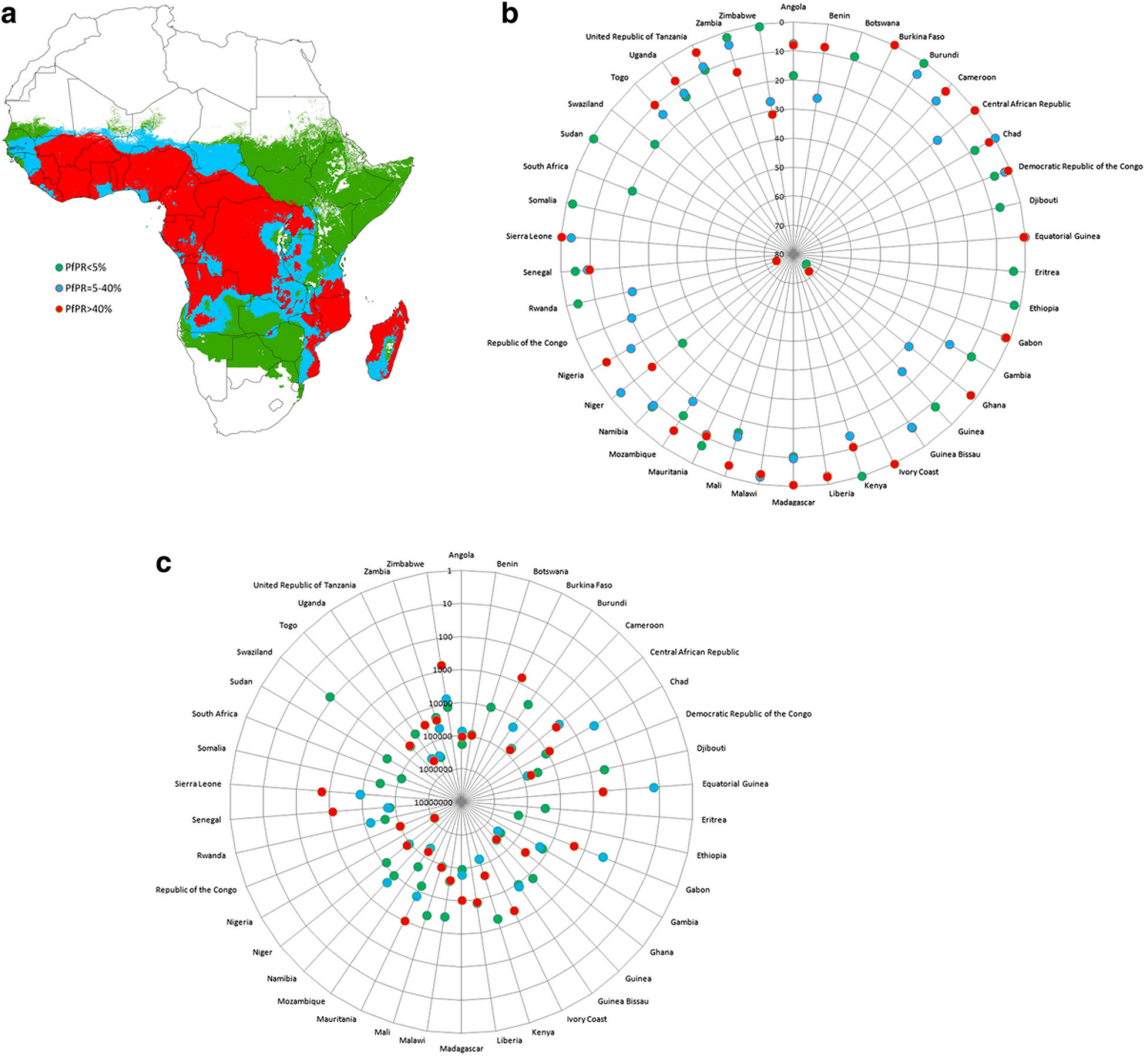
***P. falciparum *malaria prevalence in Africa and the effects on metrics of accounting for subnational age structure. (a)** Predicted prevalence classes for *P. falciparum* malaria in Africa [5]. **(b)** The absolute percentage changes in estimated numbers of children under 5 years old residing under the three prevalence classes through changing from using UN national proportions [27] to produce per grid cell estimates of numbers under 5 years to using the subnational proportion data assembled here (Additional file 1: Protocol S1). **(c)** The changes in estimated numbers of children under 5 years old residing under the three prevalence classes through changing from using UN national proportions [27] to produce per grid cell estimates of numbers under 5 years to using the subnational proportion data assembled here (Additional file 1: Protocol S1). In **(b)** and **(c)**, data values are only plotted when a transmission class encompasses >10% of the population of a country.

One component of MDG 6 is an aim to halt and begin to reverse the incidence of malaria [1], with targets focused on those under 5 years of age, upon whom the greatest burden from the disease falls. To assess achievement of these targets, and the derivation of malaria metrics in general, maps of malaria prevalence are increasingly being used in combination with spatial population datasets to estimate numbers at risk and burdens (e.g. [5,6,41,42]). The Malaria Atlas Project (http://www.map.ox.ac.uk) has recently published a mapped distribution of the intensity of *P. falciparum* transmission in 2010 based upon infection prevalence among children aged 2 to 10 years (*Pf*PR2-10) [5]. Here, the estimated distribution of prevalence by classes that have been proposed in the selection of suites of interventions at scale to reach control targets at different time periods [43,44] (Figure 2a) was used to extract estimated numbers of children under 5 years old per country residing in these different prevalence classes from (i) the AfriPop 2010 population dataset [31] adjusted to represent children under 5 using UN national proportion estimates [27] as described above, and (ii) the dataset of the 2010 population under 5 constructed from subnational data described above. For both datasets, national population totals were adjusted to match UN reported numbers [27] to ensure that any differences seen in numbers at risk were due solely to the addition of subnational information on under-5 proportions. Further details are provided in Additional file 2: Protocol S2.

Improving access to and for remote populations is an important priority for many of the MDG targets, such as those focused on eradicating extreme poverty, achieving universal primary education, and developing a global partnership for development [1]. Moreover, each health-related goal is dependent upon accessing populations to provide interventions, principally delivered through health facilities, and the difficulty in traveling to these facilities has been consistently highlighted as a barrier to treatment in rural populations, particularly in maternal health [45,46]. The measurement of accessibility or “remoteness” of populations is therefore of importance in measuring progress toward achieving these goals, and increasingly, approaches based on GIS-derived travel times are being applied [45-51]. A recently developed continent-wide travel time dataset [52,53] was used here to map those regions estimated to be greater than five hours from the nearest settlement of population size greater than 50,000. This dataset was used as an illustrative proxy for health system access, since reliable continent-wide datasets on health facility locations do not currently exist. To demonstrate the size of the variations achieved when using actual health facility data, for eight countries with open-access datasets of health facility locations (Additional file 2: Protocol S2), maps representing estimated travel times to the nearest facilities were constructed following previous approaches [50-55] (Additional file 2: Protocol S2). The accessibility datasets were used to extract estimated numbers of women of childbearing age per country residing in different travel time classes from (i) the AfriPop 2010 population dataset [31] adjusted to represent women of childbearing age using UN national proportion estimates [27] as described above and (ii) the 2010 distribution dataset of women of childbearing age constructed from subnational data described above.

## Results

### Data assembly and risk group distributions

Over a million individual data records were collected and matched to 20,381 administrative units across the continent (Figure 1a, Additional file 1: Protocol S1). The subnational proportions (Figures 1a, 1b) and growth rates (Additional file 1: Protocol S1) were combined with existing spatial population datasets [31] and UN country total estimates [27] as described above to produce high-resolution age-and sex-structured Africa-wide spatial population datasets for 2000, 2005, 2010, and 2015 (Figure 1c, Additional file 1: Protocol S1). For comparison of the effects of the inclusion of subnational age and sex structure data on health metrics, the same spatial population count dataset [31] was combined with UN Population Prospects national-level data on age and sex proportions [27], as described above, to produce alternative high-resolution Africa-wide spatial population datasets that assumed homogeneity in age and sex structures across countries.

The distributions of the proportion of children under 5 at subnational scales (Figure 1a) and women of childbearing age (Figure 1b), show the great differences that exist on a continental scale between the majority of sub-Saharan African countries and those higher-income countries in southern and northern Africa. Moreover, large variations are also seen within country borders, whether the proportions are measured at the coarsest subnational units of administrative level 1 (provincial) or as fine a detail as level 4 (wards in Tanzania). This great heterogeneity in subnational population composition across the continent is often ignored in the application of existing spatial population datasets, thus assuming demographic homogeneity across countries (e.g. [6,9,10,28,30,37]).

**Figure 3:**
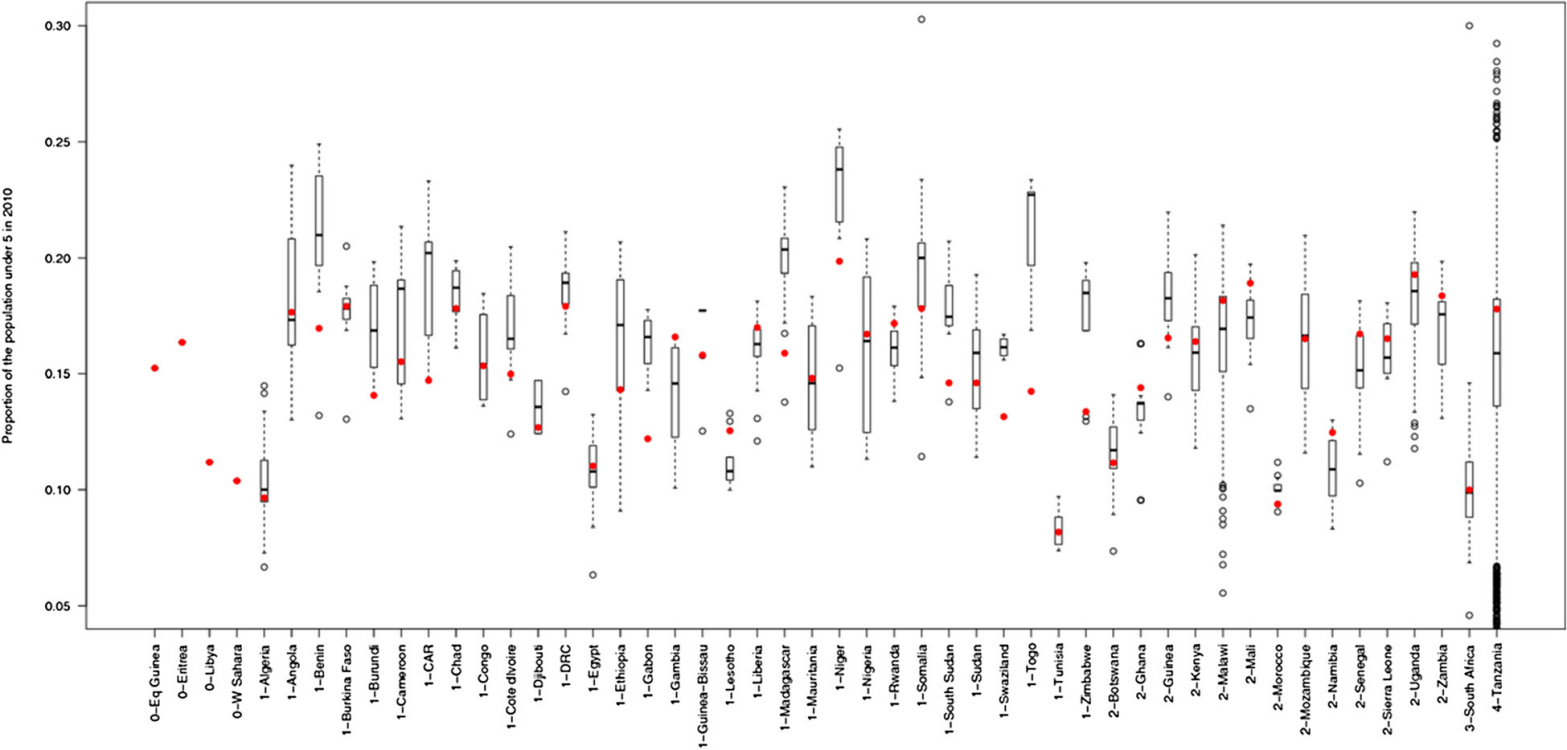
**Differences between national and subnational summaries of population proportions under 5 years old for African countries.** Comparison of UN national estimates [27] of proportions of the population under 5 years old in 2010 (red dots) against the range of proportions measured by the subnational datasets collated here (Additional file 1: Protocol S1) shown as boxplots. The solid center line of the boxplot shows the median values, the box width represents the interquartile range, and the whiskers extend to 1.5 times the interquartile range from the box (values further away than this are shown as open circles). The administrative unit level of the subnational data used here is shown as a prefix to the country name on the x-axis.

An indication of the size of subnational variation in the proportion of the population under 5 captured in the subnational level dataset (Figure 1a) that is missed through summarization to national levels is highlighted in Figure 3. The size of this variation is related to the administrative unit level of the input data, with those countries for which the most spatially detailed age structure data were available (Tanzania and South Africa) showing the largest differences between minimum and maximum estimates of per-unit proportions of residents under 5. Nevertheless, even for those countries where subnational data were only available at administrative unit level 1, differences of +/- 5% from the UN national estimates are common. Moreover, national estimates are often reflective of proportions in urban areas, where the majority of people reside, hiding the extremes that exist in rural areas, and this is evidenced by the UN estimates falling outside of the interquartile range of the boxplots in many cases (Figure 3).

### Effects on health metrics

**Figure 4:**
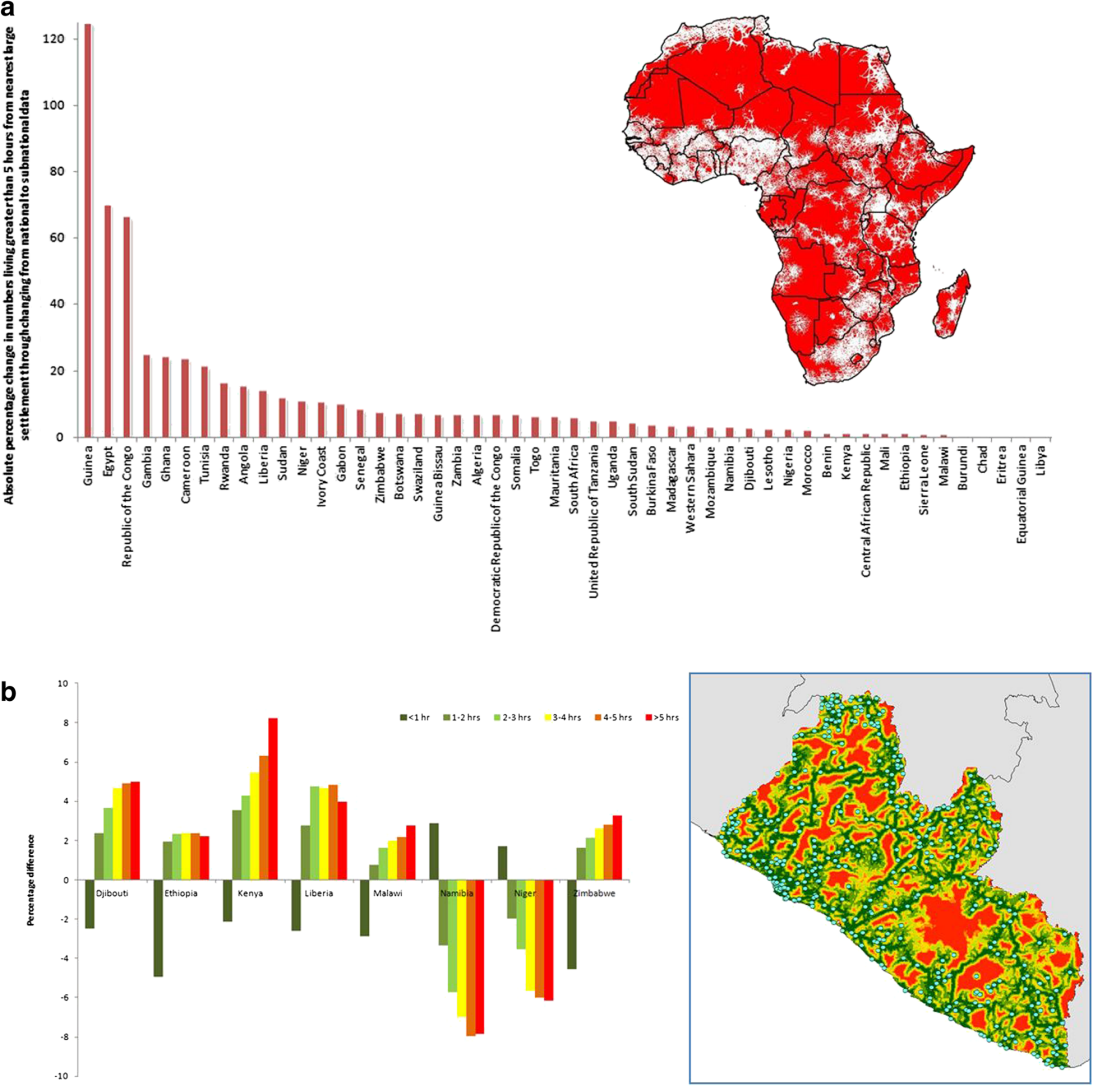
**The effects of accounting for subnational age structure on estimates of travel times to settlements and health clinics. (a)** The absolute percentage changes in estimated numbers of women of childbearing age residing greater than five hours from the nearest settlement of population size larger than 50,000 people through changing from using UN national proportions [27] to the subnational data assembled here (Additional file 1: Protocol S1). The inset map shows those areas over five hours from the nearest settlement of population size greater than 50,000 in red. **(b)** The percentage changes in estimated numbers of women of childbearing age residing at different travel times from their nearest health facility for eight countries through changing from using UN national proportions [27] to the subnational data assembled here (Additional file 1: Protocol S1). The inset map shows the modeled travel times to health facilities in Liberia using the same coloring as the bar plot.

In terms of using cartographic approaches to estimating the numbers of people in specific age groups either impacted by disease or able to access large settlements and health facilities, it is clear that the use of subnational data on age structures can result in substantial differences in output indicators over simply using national-level proportions. Figure 2 shows these differences for each country in estimates of the number of children under 5 residing under different *P. falciparum* malaria prevalence classes. The simple use of national level age structure adjustments for estimates of numbers within different age groups at risk of *P. falciparum* malaria, as undertaken in many recent studies, can result in some estimates being nearly 100% different from those obtained using more detailed data that capture subnational demographic variations, with close to half of countries exhibiting absolute differences greater than 10% (Figure 2b). For small countries, such as Guinea-Bissau, this translates to estimates changing by only a few thousand people, whereas for more populous countries, such as Nigeria, this results in many millions of children changing classes (Figure 2c). Such trends are also seen when quantifying travel times to major settlements and health facilities for women of childbearing age (Figure 4). At a continental scale, estimates of numbers residing more than five hours from a large settlement are greater than 10% different for half of the countries if an assumption of age structure homogeneity across the country is applied, compared to accounting for the subnational variations that exist (Figure 4a). Further, similar findings are evident when estimating numbers of women of childbearing age residing at different travel times from health facilities (Figure 4b), emphasizing the impact that spatial demographic heterogeneity has upon the measurement of health facility access for vulnerable populations. For six of the eight countries examined, if the proportion of the population that women of childbearing age make up is assumed to match national estimates [27] across the country, then the numbers residing at travel times of more than an hour from the nearest health facility will be consistently underestimated (Figure 4b) due to subnational variations in age and sex structures. For Namibia and Niger, the two most sparsely populated countries, the reverse is true.

## Discussion

The assessment of progress toward meeting the MDGs will be measured through national-level indicators [1] that can mask substantial inequalities across nations [12,18,56]. The development of cartographic approaches to transforming georeferenced data on health and development metrics into valuable spatial datasets is opening opportunities for quantitative assessments of these inequalities, the targeting of interventions and measurement of progress toward the MDGs, but demographic spatial datasets to support such efforts remain reliant on coarse and outdated input data for accurately locating risk groups.

While high-resolution spatial data on population distributions in resource poor areas are now becoming available (e.g., http://www.afripop.org, http://www.asiapop.org, http://www.census.gov/population/international/data/mapping/demobase.html), comprehensive and contemporary subnational information on the demographic attributes of these populations remain scattered across national statistics office reports and household surveys [19]. Here approaches to combining these publicly available disparate datasets are presented, enabling the production of Africa-wide datasets depicting age and sex compositions at subnational scales. The datasets and analyses highlight the importance of accounting for subnational demographic variations in deriving health and developments metrics. Both the large subnational variations in age and sex population structures that are evident (Figures 1 and 3), and the resulting impacts that these have on metric derivation (Figures 2 and 4) underline the need to obtain and utilize the most spatially refined data available.

The ranges of proportions of the population that is under 5 years old seen when comparing the subnational versus national-level estimates (Figure 3) highlight the need for more spatially detailed demographic data to better capture these variations. Differences of +/-5% in the proportions are common, and the spatial configuration of those areas that are substantially greater or less than the UN estimates in relation to the spatial distribution of disease risks or access, as seen in Figures 2 and 4, can have major implications on the derivation of indicators. Whilst the distributions of predicted malaria risk or travel times are mapped as continuous variables at 1km spatial resolution, if the population distribution data used to derive numbers at risk is based upon an assumption of age and sex structure homogeneity through national-level estimates, it is clear that this can result in some significant inaccuracies that consistently remain unacknowledged. Clear urban and rural differences (Additional file 1: Protocol S1) also highlight the need for accounting for such variations, and when indicators such as malaria risk or access to health facilities that vary substantially by urban-rural divides are being estimated, the large effects of this are evident (Figures 2 and 4). For example, in Kenya some of the most rural areas have the highest malaria transmission, the largest travel times to health facilities, and the highest proportions of children under 5/lowest proportions of women of childbearing age. Thus, accounting for all three of these factors subnationally compared to assuming a homogenous demographic structure results in substantial differences in outcome metrics (Figures 2 and 4). As funding for health and poverty-related mapping and the number of new cartography projects (e.g. [57-60]) continues to grow, the need for accurate spatial population distribution data will also grow if denominator-reliant metrics are required.

While accounting for subnational heterogeneity in population attributes likely results in significant improvements in the accuracy of health metrics, it is clear that many sources of uncertainty and error remain. All of the census and survey-based data used here are subject to various sources of error and bias, many of which have been well documented [61]. Indigenous groups, informal settlements, places experiencing civil unrest, and refugees are often entirely unsampled, either because of political biases, missing sampling frames, or prohibitive difficulties in carrying out a survey. Uncertainties also arise over comparisons being made between primarily census-based national estimates of age/sex proportions from the UN Population Prospects [27] and the household survey-derived subnational age/sex proportions used here for some countries. Differences between the way these proportions were measured contributes to uncertainties in comparisons between outcome health metrics, though strong correlations between the household survey-derived age structures and those derived from census data suggest that such differences may be small (Additional file 1: Protocol S1). Further, the underlying AfriPop population datasets contain uncertainties [31], while for some countries, the input data used here remains outdated and coarse (Figure 1, Additional file 1: Protocol S1). Like most other population parameters reported for administrative polygons, the age and sex proportions are also subject to the modifiable areal unit problem [62]. Discretising (by gridding) a phenomenon that is continuous (or in this case, varying at a far higher resolution) is an arbitrary process. In the case of the datasets presented here, whilst the precision with which heterogeneities in vulnerable population distributions are mapped is improved over simple national adjustments, we are still faced with a dataset containing one set of values for Libya and thousands for Tanzania. There is therefore a need to more rigorously quantify the uncertainties inherent in spatial demographic datasets. The advancement of theory, increasing availability of computation, and growing recognition of the importance of robust handling of uncertainty have all contributed to the emergence in recent years of new, sophisticated Bayesian approaches to the large-scale modeling and mapping of disease [4,7,25], but such methods have yet to cross over to the spatial demographic databases with which such maps are used. The regular availability of new national household surveys means that more contemporary data is continually becoming available to aid in updating and improving the accuracy of the datasets presented here, potentially through automated systems that can rapidly adapt to new incoming data and integrate them into the output spatial datasets, alongside robust methods to account for temporal differences [63].

The international focus on health-related goals coupled with a growing trend in research and funding for cartographic approaches to deriving metrics are increasing needs for spatial demographic data of similar scope for use in estimating denominator sizes and characteristics of populations at risk. The importance of accounting for subnational demographic variations in deriving health metrics is clear and the size of the differences that exist between ignoring subnational variations in age and sex structures, compared to accounting for them, is large enough to make the difference between success and failure in meeting a MDG. Here we have shown that sufficient data exists to produce a continent-wide subnational picture of demographic attributes and the mapping of key risk group distributions. Gridded age-structured datasets for 2000, 2005, 2010, and 2015 are freely available to download from the AfriPop project website (http://www.afripop.org) and will be regularly updated as new data become available. Similar datasets for Asia and Latin America will soon be made available through the AsiaPop (http://www.asiapop.org) and AmeriPop (http://www.ameripop.org) projects.

## Competing interests

The authors have declared that no competing interests exist.

## Authors’ contributions

AJT conceived the study, undertook data assembly and analyses, and wrote the paper. AJG, AEG, MG, and CL aided with data analyses and writing the paper. PWS and AMN aided with data collection, processing, and writing the paper. All authors read and approved the final manuscript.

## Supplementary Material

Additional file 1: Protocol S1 Constructing spatial demographic datasets for Africa. Click here for file

Additional file 2: Protocol S2 Calculating cartographically-derived health metrics. Click here for file
