# Supplementary material for: Millennium development health metrics: where do Africa’s children and women of childbearing age live?
Source: Popul Health Metr. 2013 Jul 23;11:11. doi: 10.1186/1478-7954-11-11 (PMC3724578; doi:10.1186/1478-7954-11-11)
Supplement: Additional file 1: Protocol S1 — Constructing spatial demographic datasets for Africa. [file 1478-7954-11-11-S1.doc]

**Protocol S1: Constructing spatial demographic datasets for Africa**

**Contents**

**1.1 Assembling subnational GIS boundary-linked age and sex data**

**1.2 Household survey and census microdata versus census data age-structure comparisons**

**1.3 Quantifying populations and differences by urban, peri-urban and rural settlement types**

**1.4 Assembling subnational GIS boundary-linked intercensal population growth rates**

**1.1 Assembling subnational GIS boundary-linked age and sex data**

The main paper provides information on how the GIS boundary-linked age and sex data were assembled and compiled. Here the data sources and features are documented. Table S1.1 documents the sources and features of the five-year groupings of subnational age and sex data used in construction of the 2010 population datasets described in the main paper. Where multiple data sources informing on the proportions that were residing in each unit were available, the dataset that was closest in time was chosen, with census data having priority over household survey data if available within the 2000-2010 period, due to its substantially larger sample sizes. The data for 18 of the 50 countries were derived from full national population and housing censuses, 8 from census microdata, 11 from traditional DHS household surveys, 3 from MIS and AIS, and 6 from MICS (figure S1.1). These data were matched to GIS shapefile datasets that mapped the boundaries of the subnational units. These GIS datasets were obtained from a range of sources, including online databases (www.gadm.org), DHS-specific boundaries and boundary data from national statistical offices. Where mismatches occurred with national boundaries or between units, manual corrections were made within ArcGIS 10.1. Figure S1.2 shows the boundaries of the units and each is coloured by the proportion of the total unit population at the time of survey that is used to derive the five-year age and sex proportions.

.


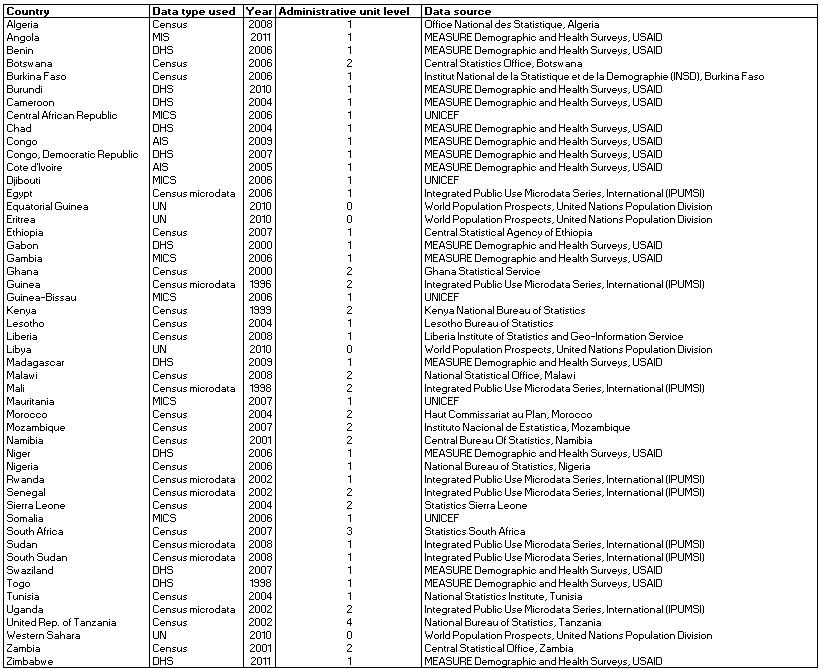


*Table S1.1. Data sources used for deriving age and gender proportions. MIS = Malaria Indicator Survey, DHS = Demographic and Health Survey, MICS = Multiple Indicator Cluster Survey, AIS = Aids Indicator Survey. Administrative unit level: level 0 = national, level 1 = provincial, level 2 = district, level 3+ = sub-district levels.*


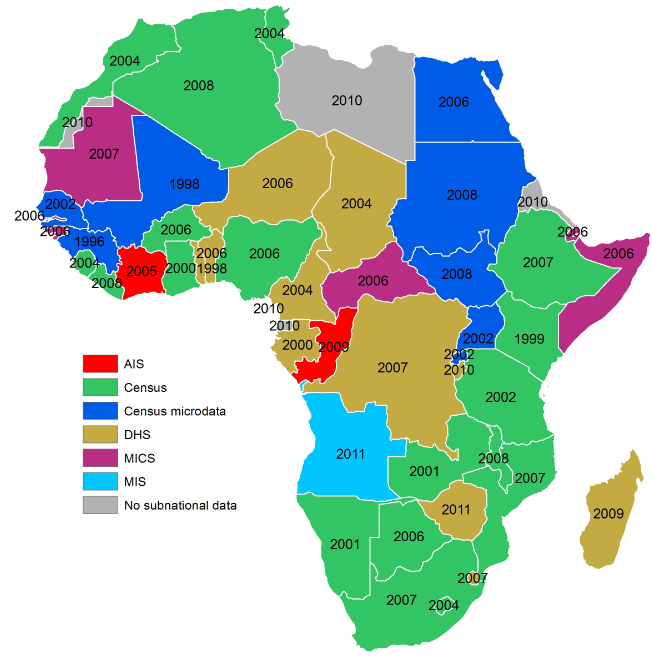


*Figure S1.1. The source and year of data used in construction of the 2010 age and sex structured gridded population datasets.*


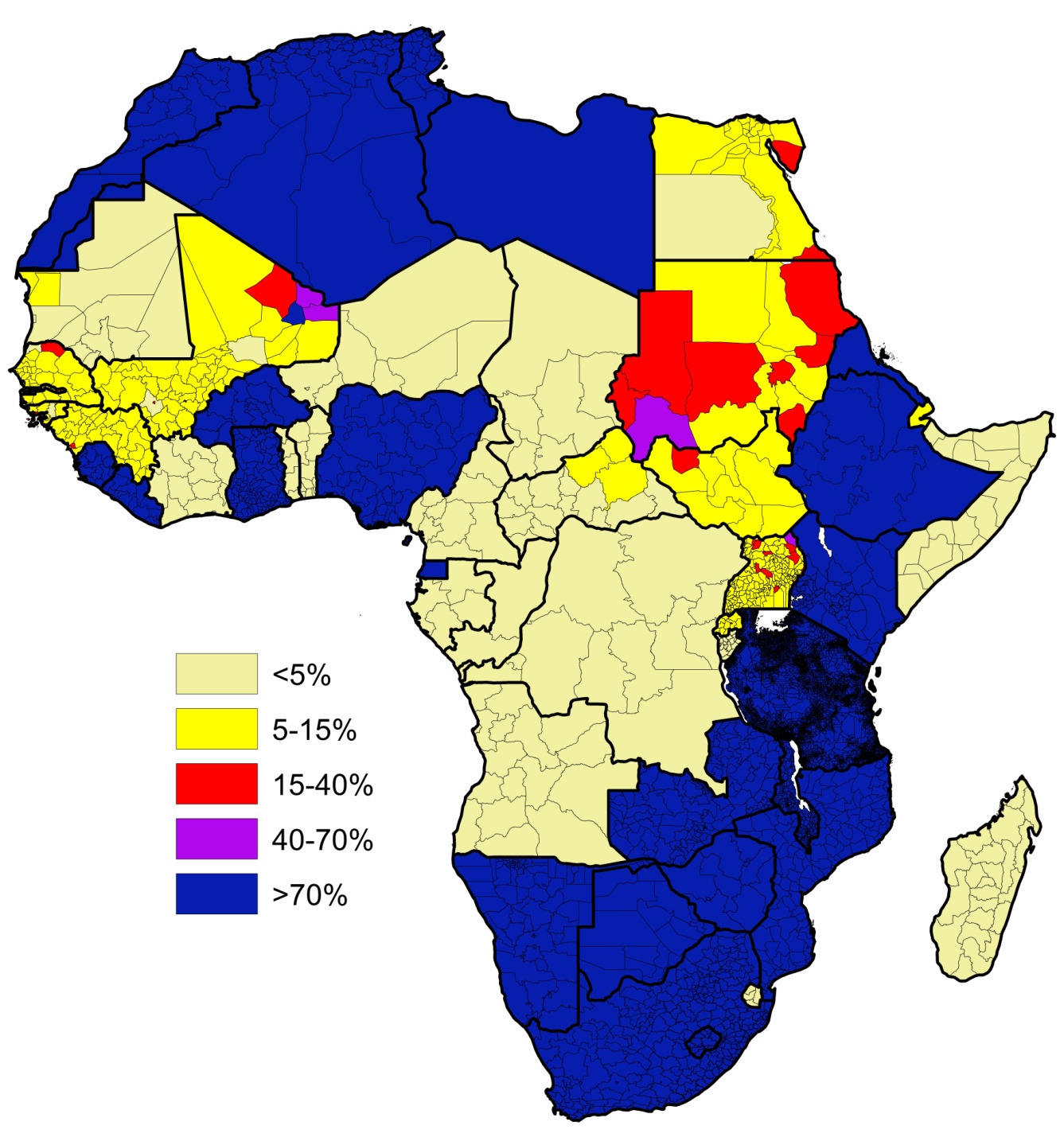


*Figure S1.2. Sample size of the subnational age and sex proportion data used, displayed as a percentage of the estimated total sub-national unit population at the time of survey.*

**1.2 Household survey and census microdata versus census data age-structure comparisons**

***Household surveys***

To test whether the small sample sizes used in household survey data produce realistic age distributions, and are therefore feasible to use in mapping at administrative unit 1 level, countries were identified where census and household survey data were collected within 1 year of each other. For each admin 1 unit, a correlation coefficient between 5-yr age proportions derived from census and from household surveys was calculated, and these were mapped out. The results for four countries are shown below in figure S3.


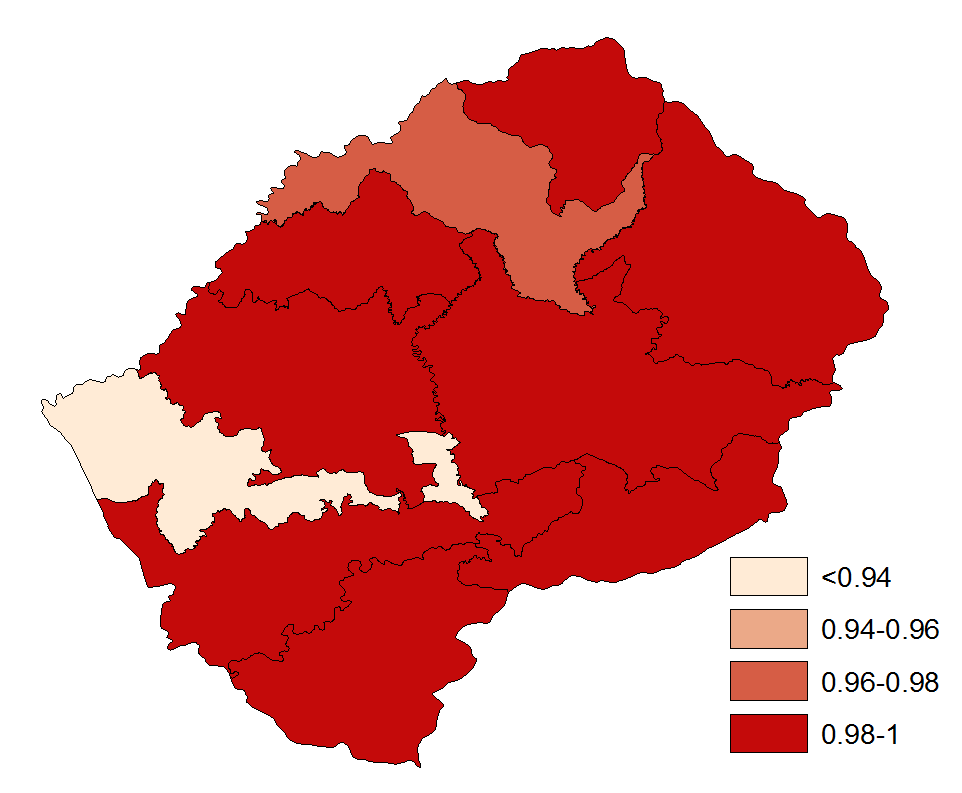

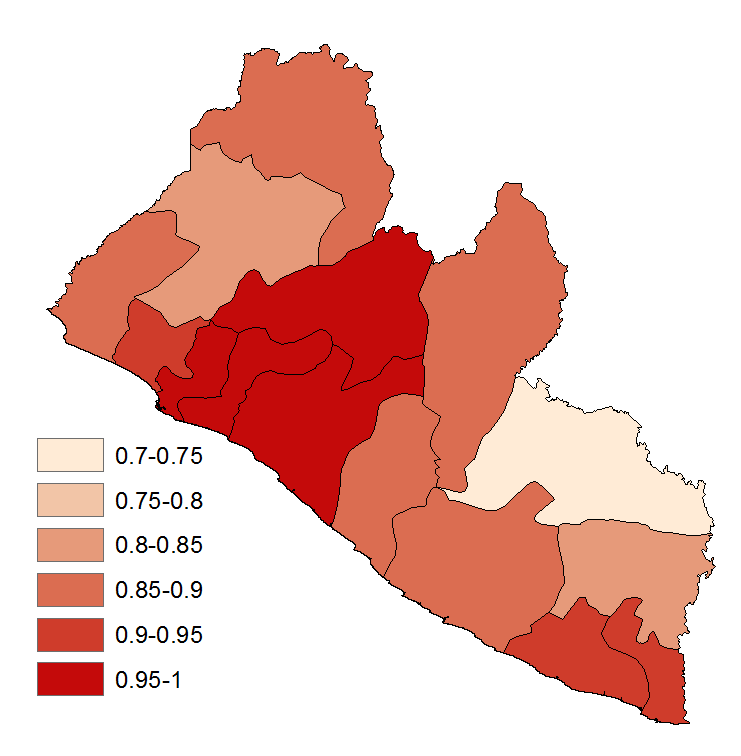


(a) (b)


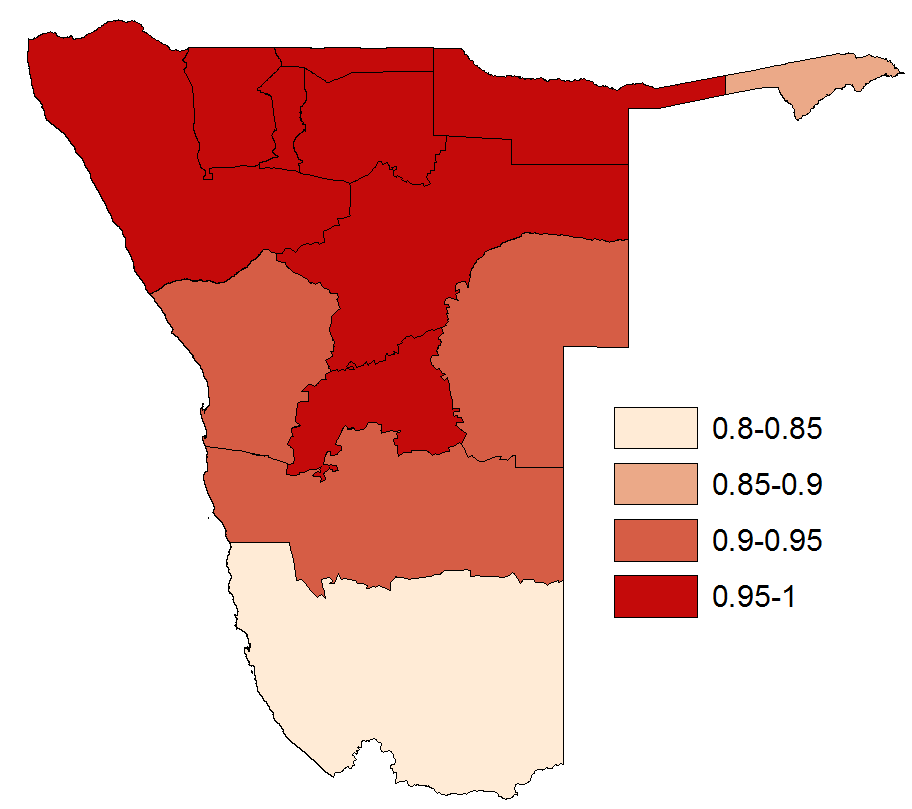

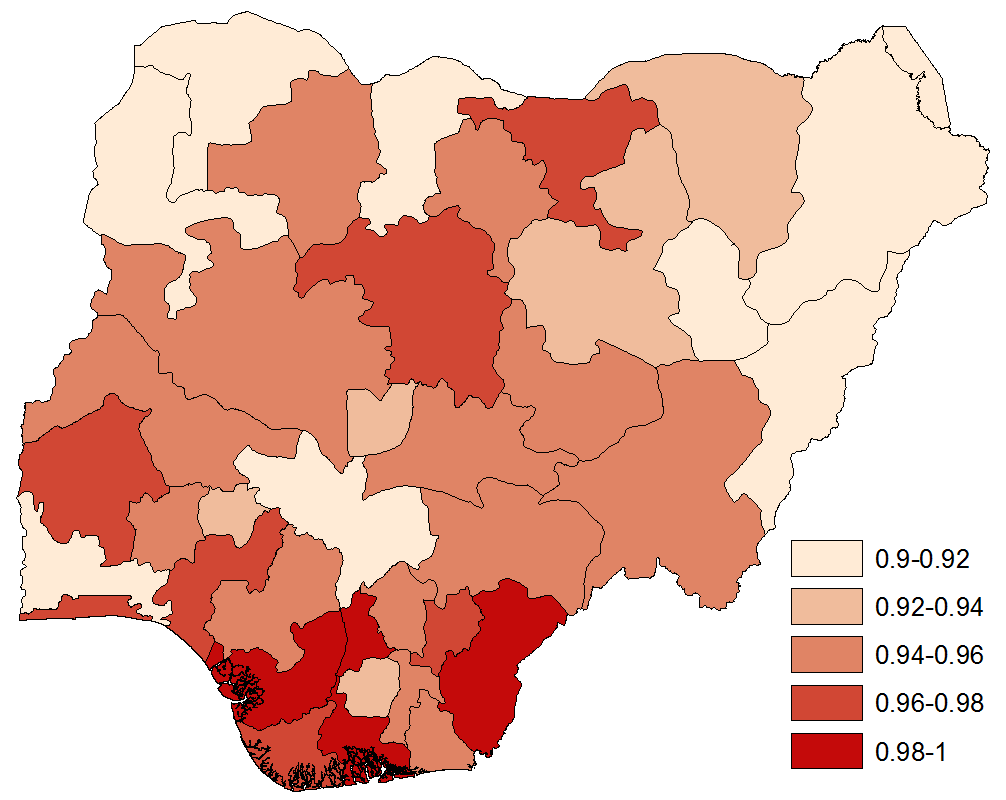


(c) (d)

*Figure S1.3. Correlation coefficients for (a) Lesotho 5-yr age group proportions derived from 2004 Census data against 2004/5 DHS data; (b) Liberia 5-yr age group proportions derived from 2008 Census data against 2008 MIS data; (c) Namibia 5-yr age group proportions derived from 2001 Census data against 2000 DHS data; (d) Nigeria 5-yr age group proportions derived from 2006 Census data against 2007 MICS data.*

***Census microdata***

To test whether the subsampling of full census data undertaken in the production of census microdata produces realistic age distributions, and are therefore feasible to use in mapping at the administrative unit level that they are available at, countries were identified where both full census and census microdata from the same census were available. For each unit, a correlation coefficient between 5-yr age proportions derived from the full census and from the census microdata was calculated, and these were mapped out. The results for five countries are shown below in figure S4.


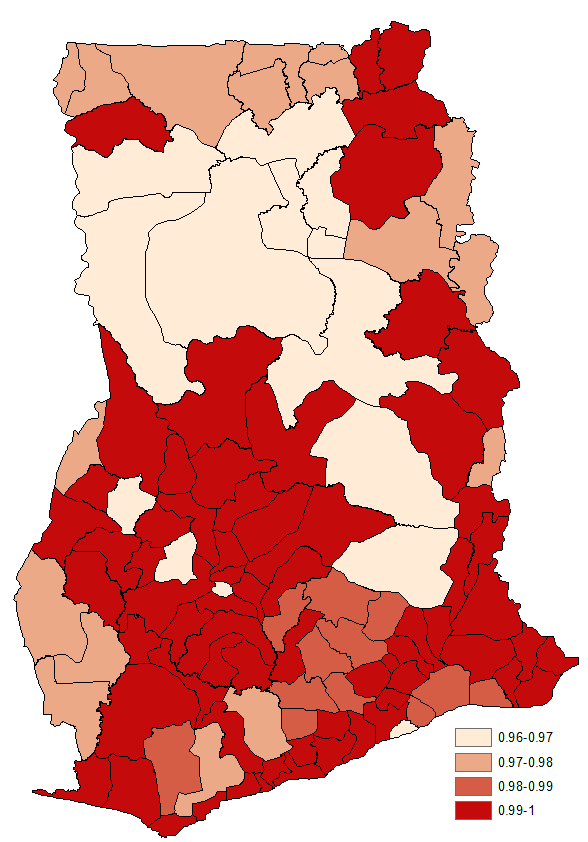

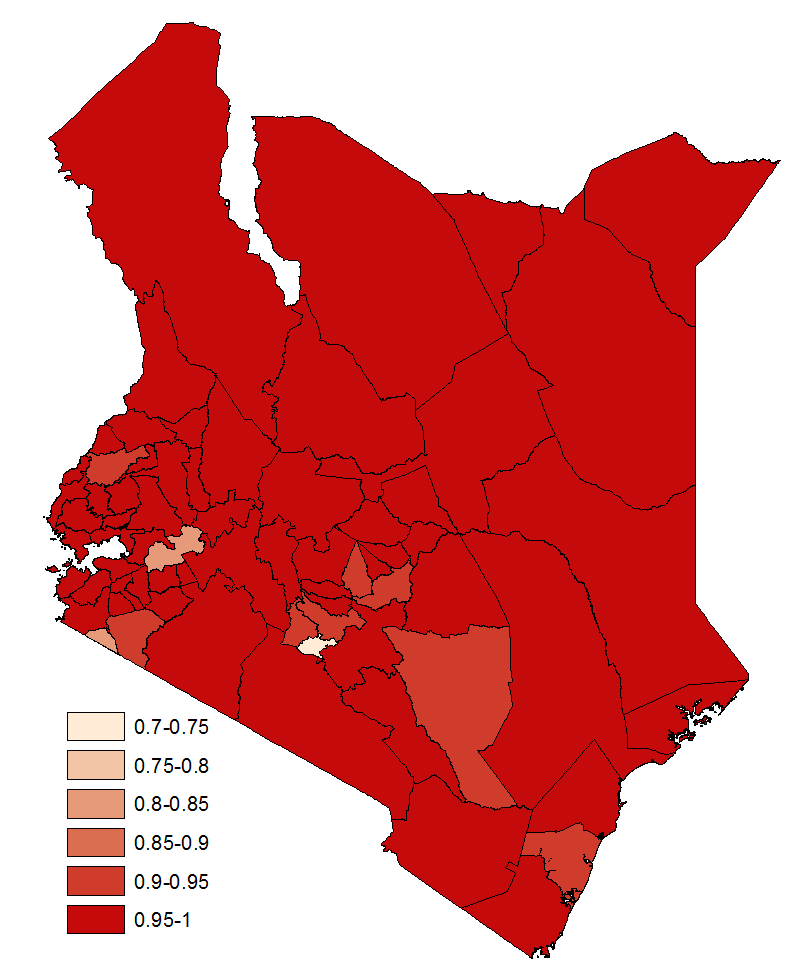


(a) (b)


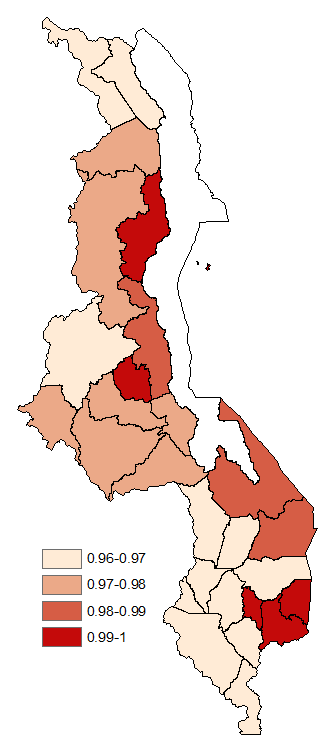

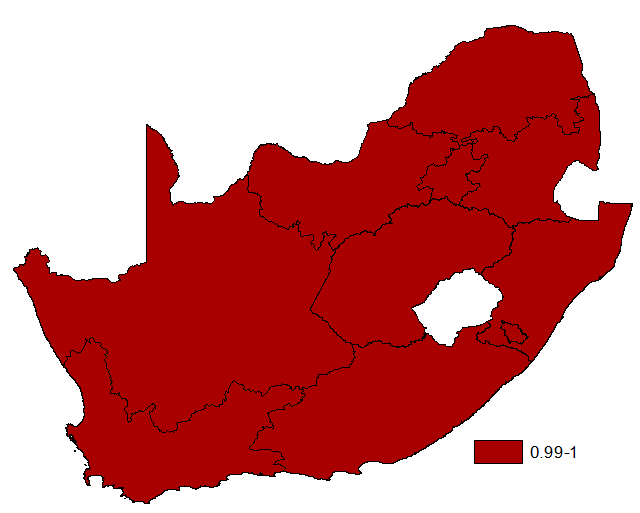


(c) (d)


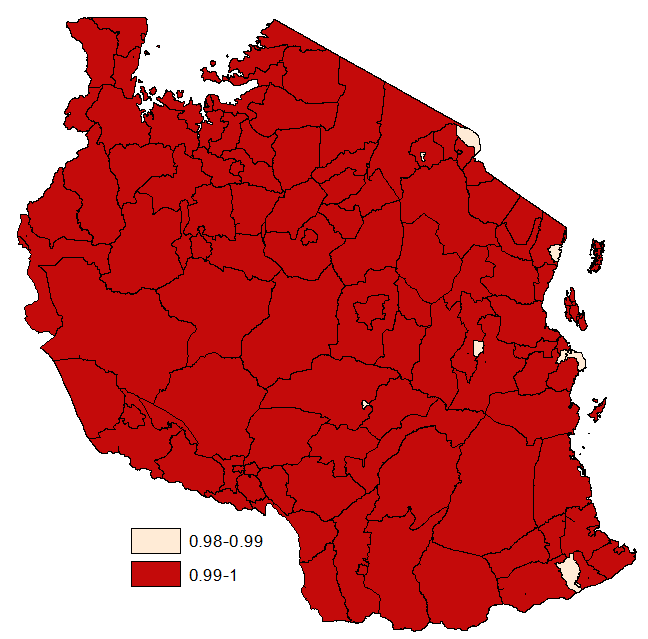


(e)

*Figure S1.4. Correlation coefficients for (a) Ghana 5-yr age group proportions derived from 2000 census data against 2000 census microdata; (b) Kenya 5-yr age group proportions derived from 1999 census data against 1999 census microdata; (c) Malawi 5-yr age group proportions derived from 2008 census data against 2008 census microdata; (d) South Africa 5-yr age group proportions derived from 2007 census data against 2007 census microdata; (e) Tanzania 5-yr age group proportions derived from 2002 census data against 2002 census microdata.*

***Summary***

Comparisons between age proportions derived from household surveys and census microdata with census data for similar time periods showed that both datasources provided a relatively accurate representation of sub-national age structures. For four countries where similar time period household survey and census data existed (Lesotho, Liberia, Namibia and Nigeria), all sub-national unit correlation coefficients were consistently above 0.7, with the vast majority well above 0.8 and showing highly significant relationships. For five countries where similar time period census microdata and full census data existed (Ghana, Kenya, Sierra Leone, South Africa, Malawi), the sub-national unit correlation coefficients were consistently above 0.95, with almost all above 0.98. These strong correlations between household survey and microcensus data-derived age proportions and those of full censuses, highlight that the comparisons undertaken in the main paper between health metrics derived using subnational versus national-level age/sex structures are likely fair ones, as the UN estimates are also based primarily on the same census data.

**1.3 Quantifying populations and differences by urban, peri-urban and rural settlement types**

The collation of sub-national information on age and sex, and its combination with AfriPop gridded population datasets meant that the numbers of people in different age groups residing in different types of settlement across the continent could be examined. Previous work has identified methods for delineating urban, peri-urban and small rural settlement extents using a combination of types of satellite imagery [1,2,3], and these are mapped in supplemental information. These settlement extents were used to extract the mean proportion and total of under 5s and women of childbearing age residing in each from the datasets described above.

The proportions of both under 5s and women of childbearing age residing in urban, peri-urban and small rural settlements by region show consistent differences, with many of these differences being statistically significant (figures S1.5 and S1.6). The transition from small rural settlements to peri-urban areas to urban areas results in a steady decline of the proportion of the population that is under 5 years old, and a steady increase in the proportion of women of childbearing age. The size of the proportions varies by region. For northern and southern Africa, overall the proportions of children under 5 years old are substantially lower than for the remainder of Africa, while women of childbearing age make up a larger proportion.


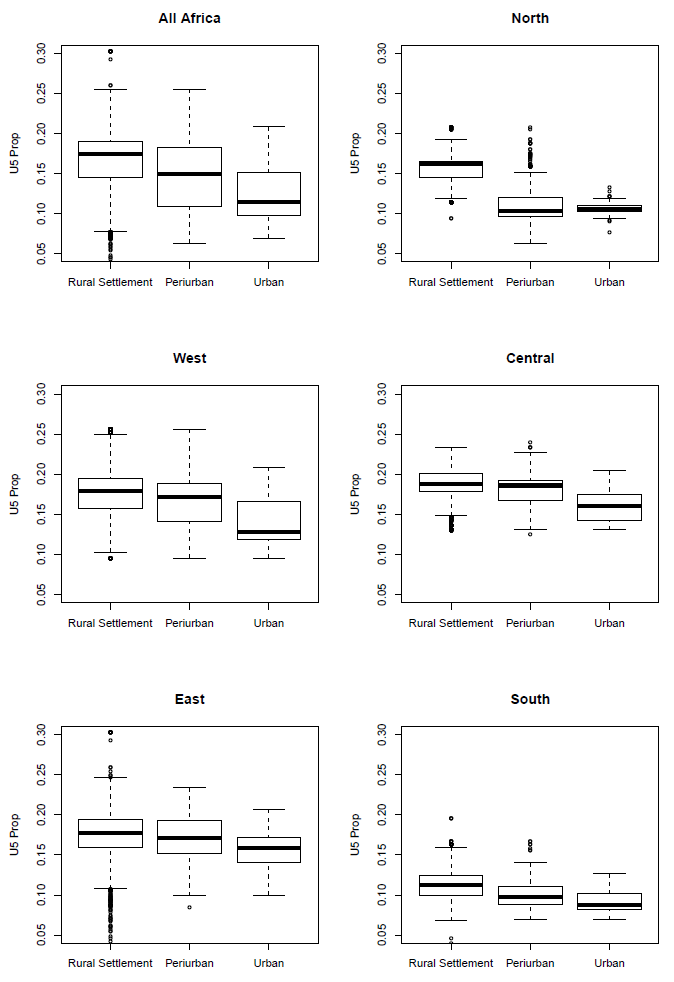


*Figure S1.5. Comparison of proportions of children under 5 years old by urban, peri-urban, rural settlement and rural class for all of Africa and broken down by UN regional groupings. The country membership of each of these groups can be found here: [http://unstats.un.org/unsd/methods/m49/m49regin.htm#africa](http://unstats.un.org/unsd/methods/m49/m49regin.htm#africa). For the ‘All Africa’, North, Central and South regions, the differences between Urban and Rural settlements were found to be significant (Mann Whitney U-test, p<0.01).*


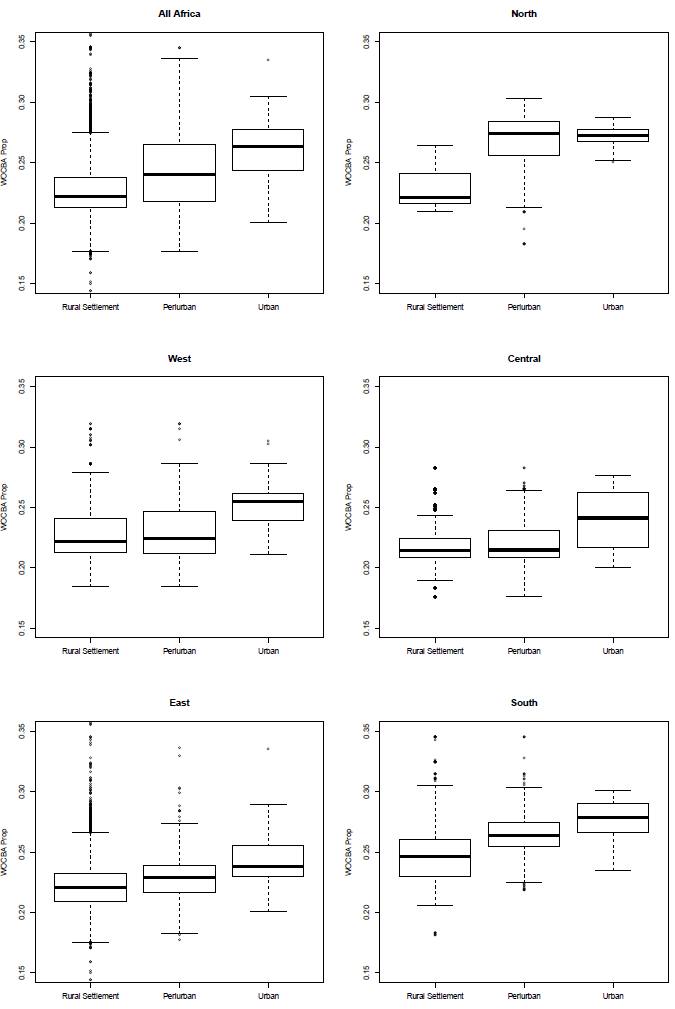


*Figure S1.6. Comparison of proportions of women of child bearing age by urban, peri-urban, rural settlement and rural class for all of Africa and broken down by UN regional groupings. The country membership of each of these groups can be found here: [http://unstats.un.org/unsd/methods/m49/m49regin.htm#africa](http://unstats.un.org/unsd/methods/m49/m49regin.htm#africa). For the ‘All Africa’, North, West and South regions, the differences between Urban and Rural settlements were found to be significant (Mann Whitney U-test, p<0.01), and for the North region the difference between Peri-urban and Rural settlements was found to be significant (Mann Whitney U-test, p<0.01).*

**1.4 Subnational GIS boundary-linked intercensal growth rates**

Sub-national growth rates from the last decade from censuses and official national estimates were collated from national statistical offices. Table S1.2 provides details of the features and sources of the datasets used, and figure S1.7 shows a map of the rates. Data on sub-national intercensal growth rates were obtained for 45 of the 50 countries, with the remaining five countries using national level growth rates [4,5]. Additionally, urban and rural growth rates and urban population estimates were obtained [5]. The combination of sub-national census administrative unit growth rates, urban-rural growth rates and urban population totals were then applied to the AfriPop 2010 population dataset [1] to obtain estimated population distribution datasets for 2000 and 2005, and these were adjusted to match UN estimates at the national, urban/rural and city level [4,5]. Finally, to produce a 2015 dataset, UN national medium-scenario urban and rural growth rates were applied [5], and the outputs were adjusted to match UN medium-scenario estimates at the national, urban/rural and city level [4,5].


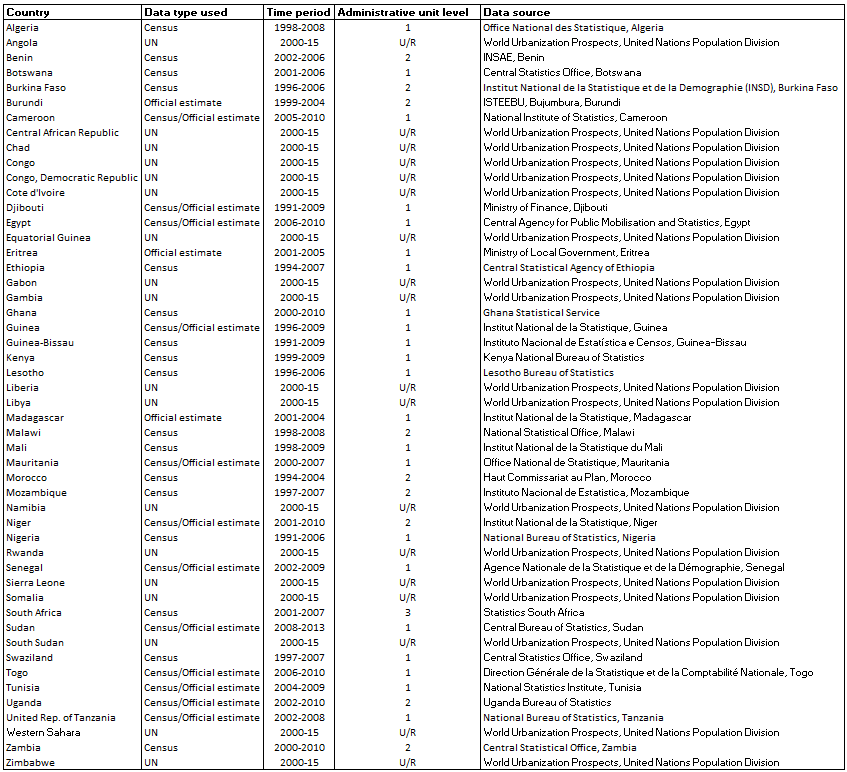


*Table S1.2. Data sources used for deriving growth rates. Administrative unit level: level 0 = national, level 1 = provincial, level 2 = district, level 3+ = sub-district levels, U/R = separate national urban and rural growth rates.*


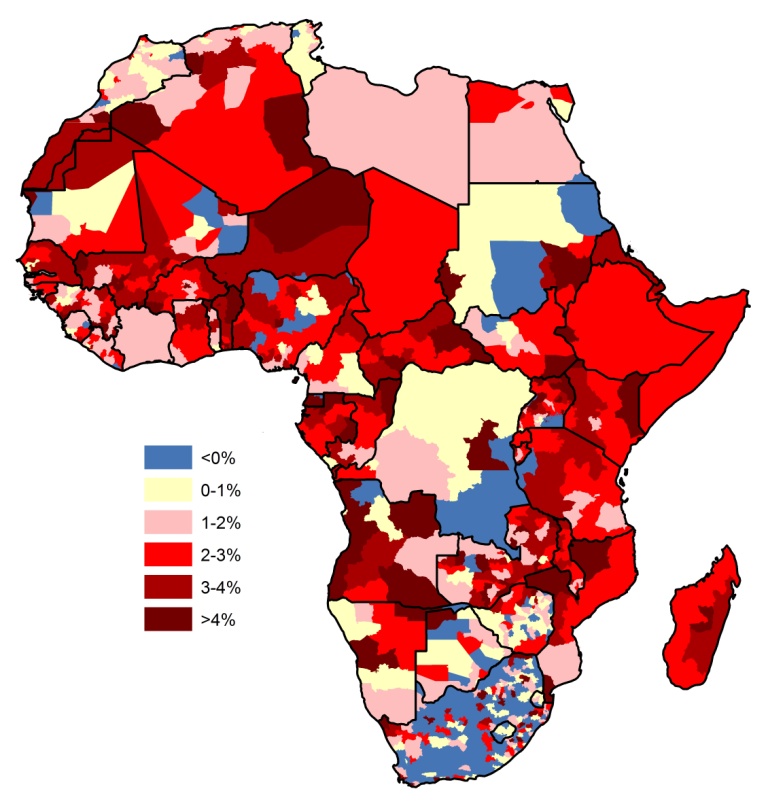


*Figure S1.7. Map showing the subnational intercensal growth rates documented in table S1.2.*

**References**

1. Linard C, Gilbert M, Snow RW, Noor AM, Tatem AJ (2012) Population distribution, settlement patterns and accessibility across Africa in 2010. PLoS ONE 7: e31743.

2. Tatem AJ, Guerra CA, Kabaria CW, Noor AM, Hay SI (2008) Human population, urban settlement patterns and their impact on *Plasmodium falciparum* malaria endemicity. Malaria Journal 7: 218.

3. Tatem AJ, Noor AM, Hay SI (2005) Assessing the accuracy of satellite derived global and national urban maps in Kenya. Remote Sensing of Environment 96: 87-97.

4. United Nations Population Division (2010) World population prospects, 2010 revision. New York: United Nations.

5. United Nations Population Division (2011) World urbanization prospects, 2011 revision. New York: United Nations.
