# Supplementary material for: Millennium development health metrics: where do Africa’s children and women of childbearing age live?
Source: Popul Health Metr. 2013 Jul 23;11:11. doi: 10.1186/1478-7954-11-11 (PMC3724578; doi:10.1186/1478-7954-11-11)
Supplement: Additional file 2: Protocol S2 — Calculating cartographically-derived health metrics. [file 1478-7954-11-11-S2.doc]

**Protocol S2: Calculating cartographically-derived health metrics**

**2.1 *P. falciparum* risk mapping**

**2.2 Estimating numbers of children under 5 at risk of *P.falciparum* transmission**

**2.3 Mapping travel times to health facilities and large settlements**

**2.4 Estimating numbers of WOCBA residing at different travel times from HFs and large settlements**

**2.1 *P. falciparum* risk mapping**

The Malaria Atlas Project has recently published revised global limits of unstable and stable *P. falciparum* infection risk and a modeled, mapped distribution of the intensity of *P. falciparum* within the stable margins of transmission based upon infection prevalence among children aged 2 to 10 years (*Pf* PR2-10) [1]. In brief, data on national case reporting, national and international medical intelligence, climate, and aridity were used to define conservatively the margins of stable and unstable *P. falciparum* transmission. Stable malaria transmission was assumed to represent a minimum average of 1 clinical case per 10,000 population per annum (pa) in a given administrative unit. Unstable malaria transmission was used to define areas where transmission was biologically plausible and/or had been documented but where incidence was likely to be less than 1 case per 10,000 population pa. In Africa, this was largely in areas where aridity limits the survival of larvae and causes desiccation of adult vectors. Finally, no transmission was assumed where assembled intelligence stated no malaria risk because (1) national reporting systems had, over several years, not reported a single *P. falciparum* clinical case, or (2) where temperatures were too low for sporogony to complete within the average lifespan of the local dominant vector species. Within the stable transmission margins, empirical community survey data on parasite prevalence were assembled and geolocated to provide the basis for an urban-rural and sample-size adjusted geospatial model within a Bayesian framework to interpolate a continuous space-time posterior prediction of *Pf* PR2-10 for every 5 × 5 km pixel for the year 2010 [1]. This model also generated classified output that assigned each pixel to one of four malaria endemicity classes: malaria-free or unstable, *Pf* PR2-10 <5%; *Pf* PR2-10 = 5% to 40%; and *Pf* PR2-10 >40% (Figure 3a in the main manuscript). These classifications of stable transmission correspond to ranges of *Pf*PR that have been proposed in the selection of suites of interventions at scale to reach control targets at different time periods [2,3].

**2.2 Estimating numbers of children under 5 at risk of *P.falciparum* transmission**

The derivation of metrics and indicators across large areas in the field of vector-borne disease epidemiology are increasingly based on cartographic approaches, whereby spatial datasets representing some disease variable of interest are overlaid on spatial datasets representing per-grid square count estimates of total numbers of people or numbers within a specific demographic group, and the total number residing within the area of interest is calculated. Such approaches are particularly prevalent in global, regional and national malaria studies [4,5].

The transmission classes mapped in figure 3a of the main paper have been used in previous studies to estimate PAR using the GRUMP dataset [4]. Here, we have examined the differences that can be obtained using alternative population datasets (Table 1). Though there exist alternative malaria risk maps [6,7], and more appropriate measures for calculating PAR that are consistent with the *P. falciparum* malaria endemicity surface used and that integrate the uncertainty inherent in the *Pf*PR2-10 estimates [8], here we compare geographical information system (GIS) overlays as done by the vast majority of previous studies [4,5] in combination with a recent *P. falciparum* dataset [1].

**2.3 Mapping travel times to large settlements and health facilities**

As discussed in the main paper, improving access to and for remote populations is an important priority for many of the Millennium Development Goal (MDG) targets, and a recent study highlighted the substantial variations that exist across Africa [9]. Here, continent-wide estimated travel time to large settlements and national level travel times to health facilities were mapped.

*Mapping travel times to large settlements*

A recently constructed dataset on global estimated travel times to settlements of population size greater than 100,000 was utilised [10,11]. Full details are provided here: <http://bioval.jrc.ec.europa.eu/products/gam/description.htm>. In brief, 'accessibility' was quantified as the travel time to a location of interest using land (road/off road) or water (navigable river, lake and ocean) based travel. This accessibility was computed using a cost-distance algorithm which computed the "cost" of travelling, in units of minutes, between two locations on a regular raster grid. The cells in this raster grid contain values which represent the cost required to travel across them, hence this raster grid is often termed a 'friction-surface'. The surface was derived from several spatial datasets that represent roads, terrain, land cover and any other geographic features that should be considered when estimating travel time to the target locations.

*Mapping travel times to health facilities*

For eight countries for which geolocated health facility data were available, travel times to the nearest facilities were mapped. Table S2.2 gives details on the travel time data. Ideally, health facility travel time analyses should account for facility type (e.g. [12]) and availability of mechanized transport (e.g. [13]), but our focus here was on examining the effects of incorporating subnational demographic information, and therefore these additions were not included in the absence of such information. Instead, travel times to the nearest facility of any types were estimated, with the assumption of mechanized transport on roads, and walking off roads.

| **Country** | **Number of facilities mapped** | **Data source** |
| --- | --- | --- |
| Djibouti | 24 | <https://gistdata.itos.uga.edu/> |
| Ethiopia | 1737 | <http://cod.humanitarianresponse.info/country-region/ethiopia> |
| Kenya | 4944 | [14] |
| Liberia | 451 | <http://cod.humanitarianresponse.info/country-region/liberia> |
| Malawi | 620 | <http://cod.humanitarianresponse.info/country-region/malawi> |
| Namibia | 338 | [15] |
| Niger | 1071 | <http://cod.humanitarianresponse.info/country-region/niger> |
| Zimbabwe | 1480 | <http://cod.humanitarianresponse.info/country-region/zimbabwe> |

*Table S2.2. Details on the geolocated health facility datasets used in analyses.*

As described above, ‘accessibility’ is a measure of friction between one location and another that takes into account land cover types, transport network and gradient. It is generally thought to be a more representative measure of ease of human travel across a landscape than simple linear distance, due to compensating for impedances to travel. Following the methods outlined above [10,11], land cover, waterbodies, slope and road network datasets were combined in a 1km spatial resolution grid and empirically-derived travel speeds [10,11] were assigned to each land use type and modified based on topography to create a ‘friction surface’ for each country. The friction surface can be converted to a map of estimated travel times to features of interest, and figure 4b in the main paper shows different travel time classes to health facility classes for Liberia.

**2.4 Estimating numbers of WOCBA residing at different travel times from large settlements and health facilities**

Following the cartographic overlay approach described in 2.2, the travel time maps for Africa and eight individual countries were thresholded to create maps of travel time classes. These were then overlaid onto the gridded women of child bearing age distribution datasets produced through (i) national level proportions, and (ii) subnational proportions, and the number of women of childbearing age living within the different travel time classes calculated.

**References**

1. Gething PW, Patil AP, Smith DL, Guerra CA, Elyazar IR, et al. (2011) A new world malaria map: Plasmodium falciparum endemicity in 2010. Malar J 10: 378.

2. Hay SI, Smith DL, Snow RW (2008) Measuring malaria endemicity from intense to interrupted transmission. Lancet Infectious Diseases 8: 369-378.

3. Smith DL, Hay SI (2009) Endemicity response timelines for *Plasmodium falciparum* elimination. Malaria Journal 8: 87.

4. Linard C, Tatem AJ (2012) Large-scale spatial population databases in infectious disease research. Int J Health Geogr 11: 7.

5. Tatem AJ, Campiz N, Gething PW, Snow RW, Linard C (2011) The effects of spatial population dataset choice on estimates of population at risk of disease. Popul Health Metr 9: 4.

6. Hay SI, Guerra CA, Gething PW, Patil AP, Tatem AJ, et al. (2009) World malaria map: *Plasmodium falciparum* endemicity in 2007. PLoS Medicine 6: e1000048.

7. Craig MH, Snow RW, le Sueur D (1999) A climate-based distribution model of malaria transmission in sub-Saharan Africa. Parasitol Today 15: 105-111.

8. Gething PW, Patil AP, Hay SI (2010) Quantifying aggregated uncertainty in *Plasmodium falciparum* malaria prevalence and populations at risk via efficient space-time geostatistical joint simulation. PLoS Computational Biology 6: e1000724.

9. Linard C, Gilbert M, Snow RW, Noor AM, Tatem AJ (2012) Population distribution, settlement patterns and accessibility across Africa in 2010. PLoS ONE 7: e31743.

10. Nelson A (2008) Estimated travel time to the nearest city of 50,000 or more people in year 2000. Ispra, Italy: Global Environment Monitoring Unit, Joint Research Centre.

11. Uchida H, Nelson A (2009) Agglomeration index: towards a new measure of urban concentration. World Development Report 2009. New York: World Bank.

12. Gabrysch S, Cousens S, Cox J, Campbell OM (2011) The influence of distance and level of care on delivery place in rural Zambia: a study of linked national data in a geographic information system. PLoS Med 8: e1000394.

13. Gething PW, Johnson FA, Frempong-Ainguah F, Nyarko P, Baschieri A, et al. (2012) Geographical access to care at birth in Ghana: a barrier to safe motherhood. Southampton, UK: Centre for Population Change.

14. Noor AM, Alegana VA, Gething PW, Snow RW (2009) A spatial national health facility database for public health sector planning in Kenya in 2008. Int J Health Geogr 8: 13.

15. MoHSS IM (2010) Namibia Health Facility Census 2009. Windhoek: MoHSS and ICF Macro. 585 p.
